# Raphe-Type Bicuspid Aortic Valve as a Risk Factor for Transcatheter Aortic Valve Replacement Failure: Improving Outcomes Using the LIRA Method and the Medtronic FX Prosthesis

**DOI:** 10.3390/jcdd12010011

**Published:** 2024-12-30

**Authors:** Francesca Napoli, Barbara Bellini, Vittorio Romano, Greca Zanda, Ciro Vella, Filippo Russo, Luca Angelo Ferri, Marco Bruno Ancona, Paolo Bonfanti, Eustachio Agricola, Antonio Esposito, Matteo Montorfano

**Affiliations:** 1 Interventional Cardiology Unit, IRCCS San Raffaele Scientific Institute, 20132 Milan, Italy; napoli.francesca@hsr.it (F.N.); romano.vittorio@hsr.it (V.R.); zanda.greca@hsr.it (G.Z.); vella.ciro@hsr.it (C.V.); russo.filippo@hsr.it (F.R.); ferri.luca@hsr.it (L.A.F.); ancona.marco@hsr.it (M.B.A.); montorfano.matteo@hsr.it (M.M.); 2 Complex Unit of Cardiology, Ospedale Valduce, 22100 Como, Italy; 3 Cardiovascular Imaging Unit, IRCCS San Raffaele Scientific Institute, 20132 Milan, Italy; agricola.eustachio@hsr.it; 4 School of Medicine, Vita Salute San Raffaele University, 20132 Milan, Italy; esposito.antonio@hsr.it; 5 Clinical and Experimental Radiology Unit, Experimental Imaging Center, IRCCS San Raffaele Scientific Institute, 20132 Milan, Italy

**Keywords:** bicuspid aortic valve, LIRA method, transcatheter aortic valve replacement

## Abstract

Transcatheter aortic valve replacement (TAVR) in patients with severe aortic stenosis and raphe-type bicuspid aortic valve (BAV) is still associated with poor outcomes in terms of increased risk of paravalvular regurgitation, stroke, and permanent pacemaker implantation. There is no definitive consensus on the optimal sizing method for prosthesis selection in this setting. The LIRA method is a supra-annular tailored sizing method specifically designed for bicuspid anatomy that might increase accuracy of prosthesis choice in BAV patients and improve TAVR outcomes. This is the first report of the combination of the novel LIRA method for prosthesis sizing together with the adoption of the technological improvements introduced by the Evolut FX prosthesis as a useful tool for improving outcomes in this high risk subgroup of patients.

## 1. Introduction

Transcatheter aortic valve replacement (TAVR) is an established treatment of severe symptomatic aortic stenosis in patients older than 75 years, according to current European guidelines [1]. The expanding indication also in younger individuals [2,3,4,5] advocates the optimization of TAVR outcomes even in patients with bicuspid aortic valve (BAV) disease.

In this setting, the TAVR procedure is more challenging due to several anatomical characteristics. These include heavily calcified leaflets, the possible presence of a raphe, which might affect prosthesis deployment, and greater aortic angulation [6].

Previous observational studies have reported higher rates of paravalvular leak (PVL), device under expansion, permanent pacemaker (PM) implantation, annulus rupture, and potentially accelerated leaflet degeneration after TAVR in patients with a bicuspid valve [7,8].

Moreover, there is still no consensus on the optimal sizing method for prosthesis selection in this population.

Recent findings have demonstrated that in raphe-type BAV anatomy, THV anchoring and sealing might occur at the level of the maximum protrusion of the raphe (that we defined as the LIRA plane) [9]. Thus, we developed a dedicated supra-annular THV sizing method (the LIRA method) that consists in the measurement of the perimeter at the LIRA plane. Indeed, the perimeter at this level gives an accurate prediction of the perimeter that will be occupied by the THV [10].

This method has proven to be highly reproducible, safe, and effective in a small group of 20 patients with BAV [10] and in a larger cohort of 50 patients [11] undergoing TAVR with various self-expanding supra-annular THV platforms (Medtronic Evolut R, Pro and Pro+; Accurate and Acurate Neo 2, Boston Scientific).

Given the compelling need to provide a safe and effective alternative to surgery, valve technologies have continually evolved and improved, leading to the development of new-generation THV platforms.

Medtronic’s supra-annular, self-expanding TAVR platform, introduced in 2007, has undergone significant advancements. Key innovations include repositioning capability during deployment (Evolut R), a porcine pericardial wrap to minimize paravalvular leak (Evolut PRO), and a smaller delivery profile for improved vascular access (Evolut PRO+). The latest generation, Evolut FX, features a flexible delivery system, radiopaque markers for enhanced positioning and commissural alignment, and an optimized stability layer for predictable deployment (Table 1).

The impact of these modifications was clinically evaluated in patients with tricuspid aortic valves undergoing TAVR. In this context, Khera et al. reported their experience with the first 43 cases of the Evolut FX and found lower PM rates and a more symmetrical deployment compared to the Evolut PRO+ [12]. Recently Zaid et al. reported the first multi-center experience of 226 patients undergoing TAVR with the Evolut FX system and compared it with a single-center PRO+ experience [13]. In comparison to the Evolut PRO+, FX has a more coaxial deployment, fewer device recaptures, improved commissural alignment, and lower rates of PVL while maintaining similar hemodynamic performance.

The main studies comparing the outcomes of the Evolut Fx with its predecessor are summarized in Table 2.

The impact of the technological improvements of the Evolut FX in BAV disease has not yet been explored.

This is the first report on the procedural outcomes of patients with BAV treated with the new Evolut FX prosthesis sized according to the innovative LIRA method.

## 2. Methods

### 2.1. Baseline Characteristics

Between June 2024 and August 2024, five patients with symptomatic severe aortic stenosis and BAV anatomy underwent TAVR with the implantation of the Evolut FX (Medtronic, Minneapolis, USA) at our institution and were enrolled in this study.

**Figure 1:**
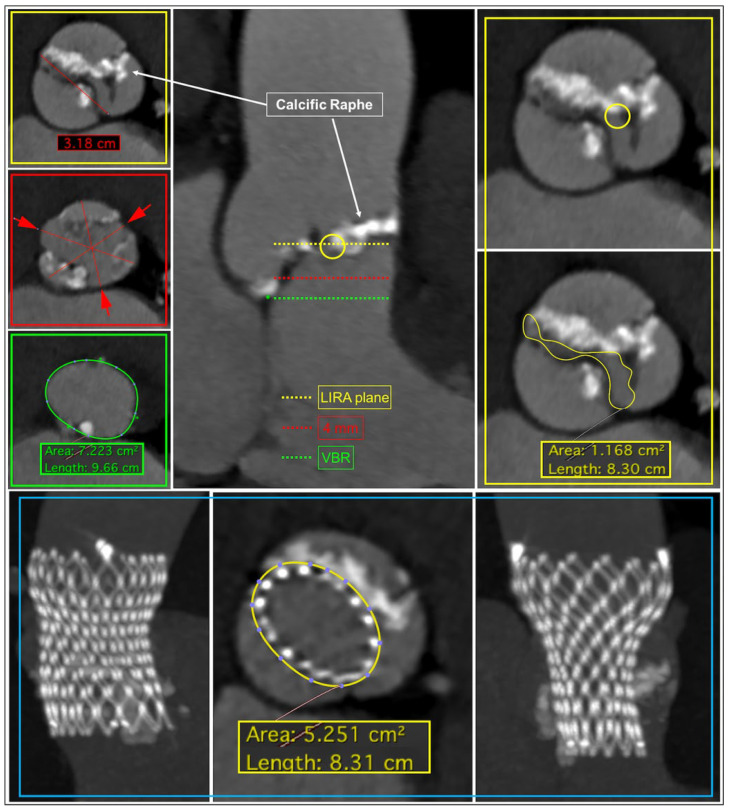
Illustration of the application of the LIRA sizing method. The upper central panel reveals the LIRA plane. It is identified as the supra-annular plane that cuts the raphe at its maximum protrusion along the aortic root, where THV anchoring, and sealing are expected to occur during deployment (yellow dotted line in the sagittal view). Notably, this supra-annular plane is not arbitrarily located at a fixed distance above the VBR, as its location differs in each patient according to the level of the raphe. The yellow circle underlines the presence of a calcific raphe between the right and the left cusp. On the left (in the upper panel) in red, the intercommissural distance at the level of the LIRA plane is displayed. Notably, ICD is not measurable at 4 mm (mid panel on the left). The green circle in the lower panel represents the perimeter at VBR. The LIRA sizing method is based on the measurement of the perimeter at the level of the LIRA plane tracing the internal border of the leaflets (yellow continued line). This perimeter predicts the perimeter that will be occupied by the THV after the implantation.

All patients underwent pre-procedural transthoracic echocardiography (TTE) and an electrocardiography (EKG)-gated cardiac CT (Computed Tomography) scan. The anatomy of the aortic valve was assessed, and BAV type was categorized according to the Sievers classification. The VBR was identified as the plane connecting the hinge points of the aortic leaflets, and measurements of the diameter, the perimeter, and the perimeter-derived diameter were obtained at this level. The LIRA plane was determined as the plane of the raphe’s maximum protrusion along the aortic root (representing the expected level of prosthesis anchoring and sealing), and measurements of the perimeter and the perimeter-derived diameter were taken at this level. The perimeter was measured by tracing the internal border of the leaflets, excluding all the structures encountered at this level, as previously described (10). In case of a discrepancy between the perimeter measured at the VBR and at the LIRA plane, the smallest value was chosen for prosthesis sizing (Figure 1). A tapered anatomy was defined in the case of a LIRA perimeter smaller than the VBR perimeter.

All patients underwent TAVR after the Heart Team discussion and signed a written informed consent for the procedure. This study was approved by the local institutional review board and conducted in accordance with the declaration of Helsinki.

### 2.2. Procedural Characteristics

All TAVR procedures were performed via a transfemoral approach under conscious sedation.

The inflow of the Evolut FX THV was aligned at the level of the annular plane at the non coronary cusp (NCC) during initial deployment in a cusp-overlap view. Before device release in the Left Anterior Oblique (LAO) projection, the 3 dot markers were aligned to remove the inflow parallax to assess pre-release implant depth at the left coronary cusp (LCC).

Pre-dilatation was performed in all patients. Decision to perform balloon post-dilatation and device recapture/repositioning was at the discretion of the implanting team.

### 2.3. Outcomes

The primary outcome of this study was the device success at 30-day follow-up, as defined by the Valve Academic Research Consortium-3 (VARC-3) criteria [16]. Secondary outcomes were technical success and intra-hospital safety outcomes. Moreover, we evaluated the relative effectiveness of the Evolut FX in achieving a more stable and predictable implant over older generations of devices by comparing the position of the prosthesis during the early phase of the deployment with its final position.

### 2.4. Statistical Analysis

Categorical variables were presented as numbers (percentages) of patients.

For continuous variables, the normality of the distribution was evaluated using the Kolmogorov–Smirnov test. Normally distributed continuous variables were expressed as mean ± standard deviation; non-normally distributed continuous variables were presented as median and inter-quartile range (IQR, 25–75th percentile).

A paired *t*-test was used to test for differences in means with continuous variables, and a two-sided *p*-value < 0.05 was considered statistically significant.

All the analyses were performed using the software STATA version 18.0.

## 3. Results

### 3.1. Baseline Clinical, Echocardiographic, and CT Scan Characteristics

The baseline clinical characteristics of this study population are reported in Table 3. The mean age was 80.4 ± 4.3, and 100% were male. The mean Society of Thoracic Surgeons (STS) Predicted Risk of Mortality was 4% ± 2, depicting a low-intermediate surgical risk population. One patient showed first-degree atrioventricular block (AVB) combined with right bundle branch block (RBBB) at the baseline EKG, both features indicating a high risk for permanent pacemaker implantation (PPI) [17].

Table 4 and Table 5 summarize pre-procedural echocardiographic and CT scan findings.

The mean left ventricular ejection fraction (LVEF) was 52% ± 15 mmHg, and only one patient presented with low-flow low-gradient aortic stenosis. CT scan analysis identified a type-1 BAV (according to Sievers classification) with raphe located in the right/left coronary position in 100% of cases. In all patients it was calcific.

All patients exhibited a mildly dilated ascending aorta, and in three patients a horizontal root was found.

The LIRA perimeter was significantly smaller than the VBR perimeter (81.04 ± 4.9 vs. 93.7 ± 4.6; *p* = 0.002).

Indeed, we found a tapered anatomy in all patients, and the application of the LIRA method led to a downsizing in all cases.

Table 2 and Table 3 summarize pre-procedural echocardiographic and CT scan findings.

### 3.2. Procedural Characteristics

Procedural characteristics are reported in Table 6.

Pre-dilatation was performed in all cases with a median balloon size of 23 (22–24) mm. Post-dilatation was required in three cases (60%).

**Figure 2:**
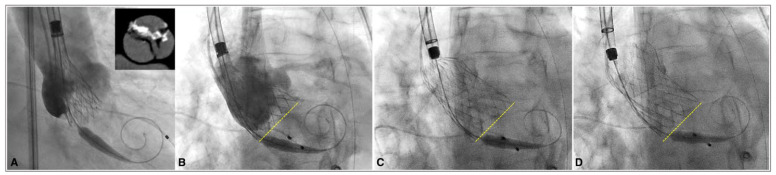
Angiographic view: comparison of initial and final position during deployment phase. Patient with type-1 BAV anatomy assessed by CT scan and implanted with a 34 mm Corevalve Evolut FX valve (**A**). The implantation height was determined using the cusp-overlap projection (**B**), and the deployment proceeded in a LAO view, ensuring the elimination of prosthesis parallax (**B**–**D**). It is important to note that the prosthesis maintains a consistent depth and orientation throughout all deployment phases, as illustrated by the yellow dotted line.

All THVs were successfully implanted, and a more predictable deployment with no ventricular movement during the initial 80% of deployment was observed, as evidenced by the valve’s stable position before and after deployment (Figure 2).

### 3.3. Clinical Outcomes

30-day clinical outcomes are represented in Table 7.

Device success and technical success, as defined by VARC-3 criteria, were achieved in 100% of cases, with no occurrence of mortality or stroke, no mean gradient ≥20 mmHg, and no moderate or severe PVL. The safety VARC-3 endpoint was met in four patients, as one required pacemaker implantation. Finally, no vascular or access-related complications were reported.

## 4. Discussion

The present study provides the first description of the clinical and procedural outcomes in patients with BAV undergoing TAVR with the new Evolut FX THV sized according to the LIRA sizing method.

The key findings of our study are as follows:

(1) TAVR with the FX prosthesis sized according to the LIRA method showed excellent procedural outcomes;
(2) The Evolut FX valve allowed stable implantation with consistent depth and orientation throughout all deployment phases.

TAVR in the setting of BAV disease still presents suboptimal results with increased mortality in the case of high-risk anatomical characteristics such as calcified raphe and excessive leaflets calcification [18] and a non-negligible rate of stroke [19,20], significant paravalvular leaks, and pacemaker implantation [19,20,21].

**Figure 3:**
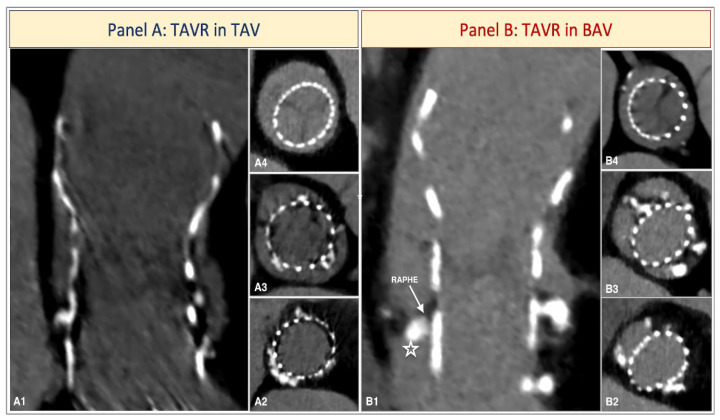
Comparison of prosthesis anchoring in tricuspid aortic valve (TAV) and bicuspid aortic valve (BAV). (**Panel A**): Post-TAVR CT scan of a 34 mm CoreValve Evolut R in a patient with tricuspid aortic valve. The long-axis view (**A1**) shows adequate expansion of the prosthesis, which appears circular in all planes displayed in the short-axis views: at the VBR (**A2**), where anchoring occurs through a 360-degree interaction between the prosthesis and the aortic annulus; at sinus plane (**A3**); at coaptation leaflet plane (**A4**). (**Panel B**): Post-TAVR CT scan of a 26 mm Corevalve Evolut R in patient with a bicuspid aortic valve. The long-axis view (**B1**) reveals that the prosthesis does not fully interact with the LVOT (white star) but instead anchors at the raphe. This observation is confirmed in the short-axis views, where the prosthesis only partially adheres to the VBR (**B2**), while it anchors at the level of the raphe and is moved towards the opposite commissure (**B3**). In both **B2**,**B3** short-axis views, the prosthesis has an elliptical shape; however, at the coaptation leaflet plane (**B4**), it gains a proper circularity, not hampering prosthesis’ performance.

With the application of the LIRA method, a tailored prosthesis sizing method specifically designed for patients with BAV, we achieved 100% technical and device success with an excellent valve hemodynamic (mean gradient of 5.8 mmHg and no patient showing significant PVL). Indeed, the use of SEV with a supra-annular design, even in the presence of frame ellipticity at the VBR and at the raphe level, allows an adequate circularity at the leaflets’ coaptation, preserving valve performance (Figure 3).

Moreover, despite the presence of a calcific raphe, a marker of worse outcomes, in all patients, we observed no mortality or stroke. The use of undersized balloons to perform pre-dilatation (sized according to the LIRA perimeter-derived diameter) might explain the low rate of stroke in our cohort.

Moreover, the application of the LIRA method in tapered anatomy (LIRA perimeter smaller than VBR perimeter) will avoid a dangerous oversizing that, especially with the implantation of balloon-expandable valves (BEV), might lead to catastrophic consequences such as annular rupture.

Finally, only 1 patient with extremely high-risk electrocardiographic characteristics (first-degree AV block and right bundle branch block) was implanted with a permanent pacemaker. In our previous studies [10,11] on the application of the LIRA method, we report a very low rate of PPI. Indeed, the undersized self-expanding valve (SEV) will anchor at the raphe level with poor interaction with the VBR. It is reasonable to speculate in future studies a possible lower PM implantation rate with the use of the novel Medtronic FX given the stable implant that allows a predictable implantation depth.

Even with the use of second-generation prostheses, in patients with raphe-type BAV, a coaxial and stable implant remains challenging [21]. Achieving a stable implant in this setting is important for two reasons:

(1) The first is the complex nature of anchoring in BAV compared to the tricuspid aortic valve [22].

Indeed, in tricuspid aortic valve (TAV) anatomies, anchoring is co-planar and occurs along 360 degrees at the VBR, thereby minimizing the impact of imperfect coaxial deployment. Conversely, in BAV anatomies, anchoring occurs primarily at the raphe. During prosthesis deployment, the driving forces exerted by the raphe can result in a prosthesis displacement in the opposite direction of the conjoint cusp with an asymmetrical anchoring of the THV on different planes. (Figure 3).

(2) Secondly, the depth of implantation is crucial in any TAVR procedure. It is well known that a low implantation depth can increase the rate of permanent pacemaker implantation due to the contact of the prosthesis frame with the membranous septum. However, a ground zero or higher implantation might preclude a safe post-dilatation due to the high risk of migration and embolization [23]. In patients with TAV, anchoring occurs at VBR, and it is guaranteed by the prosthesis’ oversizing so that implantation depth in this setting is standardized and highly predictable. Conversely, in patients with BAV, the implantation depth is influenced by parameters that vary among patients, such as the orientation and particularly the height of the raphe. In this context, the waist and the shape of the prosthesis at the deployment represent a fundamental indicator of implantation depth, and specific configurations of the THV might be associated with an increased risk of embolization in case of post-dilatation.

**Figure 4:**
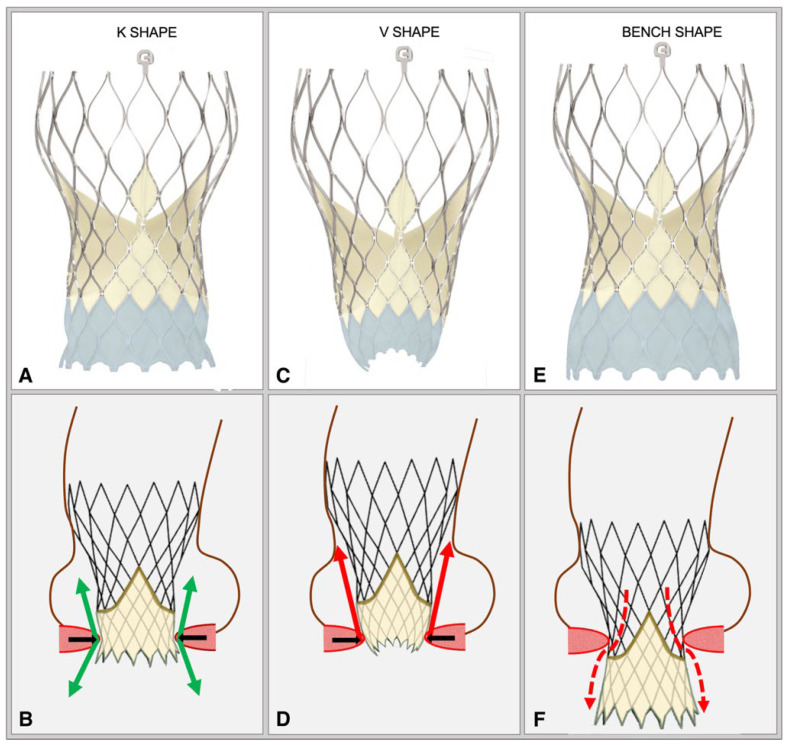
Various configurations of prosthesis’ shape at deployment. The implantation depth influences the extent of deformation of the inflow portion (light blue area in the first row: **A**,**C**,**E**) of the prosthesis, resulting in three different configurations: **K shape** (**A**,**B**): the deformation of the landing zone shows a K-shaped neck (**A**). The green arrows (**B**) indicate the two opposite and balanced forces (downward toward the LVOT and upward toward the ascending aorta) that ensure the stability of the prosthesis. These forces are generated by the interaction (**black arrows**) between the prosthetic stent and the aortic valvar complex. **V shape** (**C**,**D**): the deformation of the landing zone shows a V-shaped appearance (**C**), with the prosthetic diameter being smaller at the level of the prosthetic inflow. In this case (**D**), the prosthesis is implanted too high, and the VBR (**black arrows)** generates forces directed mainly upwards (**red arrows**), increasing the risk of embolization in case of post-dilatation. **Bench shape** (**E**,**F**): the landing zone does not show significant deformations (**E**), and it is entirely within the left ventricle, creating a direct communication (**red arrows**) between the prosthetic outflow and the VBR (**F**). In this case (**F**), the prosthesis is implanted too low, resulting in a modest interaction between the prosthetic frame and the VBR.

**Figure 5:**
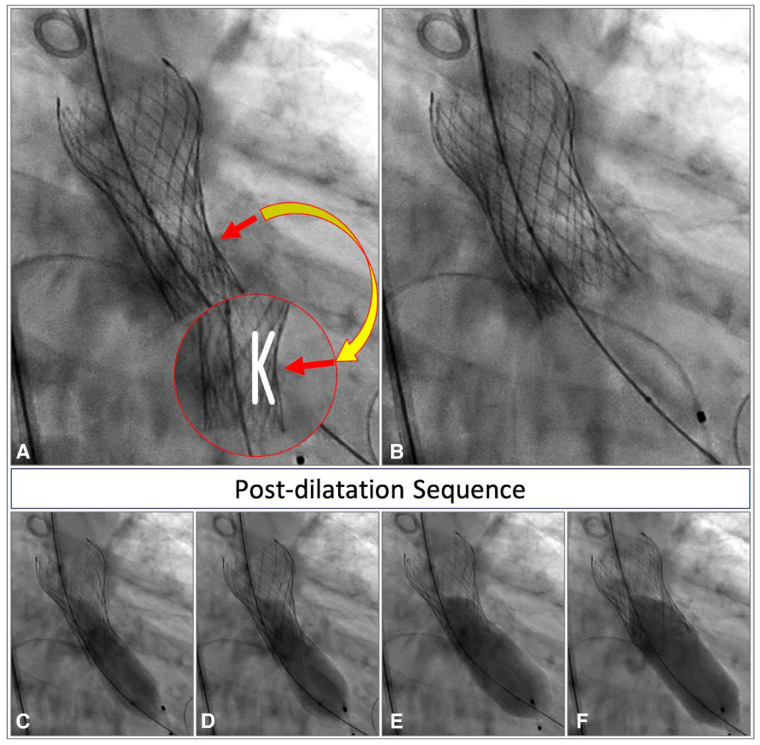
Angiographic view of a Corevalve Evolut R with “K” shape at deployment and its post-dilatation sequence. (**A**) Under-expanded Corevalve Evolut R in the presence of leaflet calcification, causing a waist in its inflow portion (red arrow) with a K-shaped appearance at implantation (red circle, magnified view); (**B**) optimal final result after post-dilatation; (**C**–**F**) safe post-dilatation sequence.

**Figure 6:**
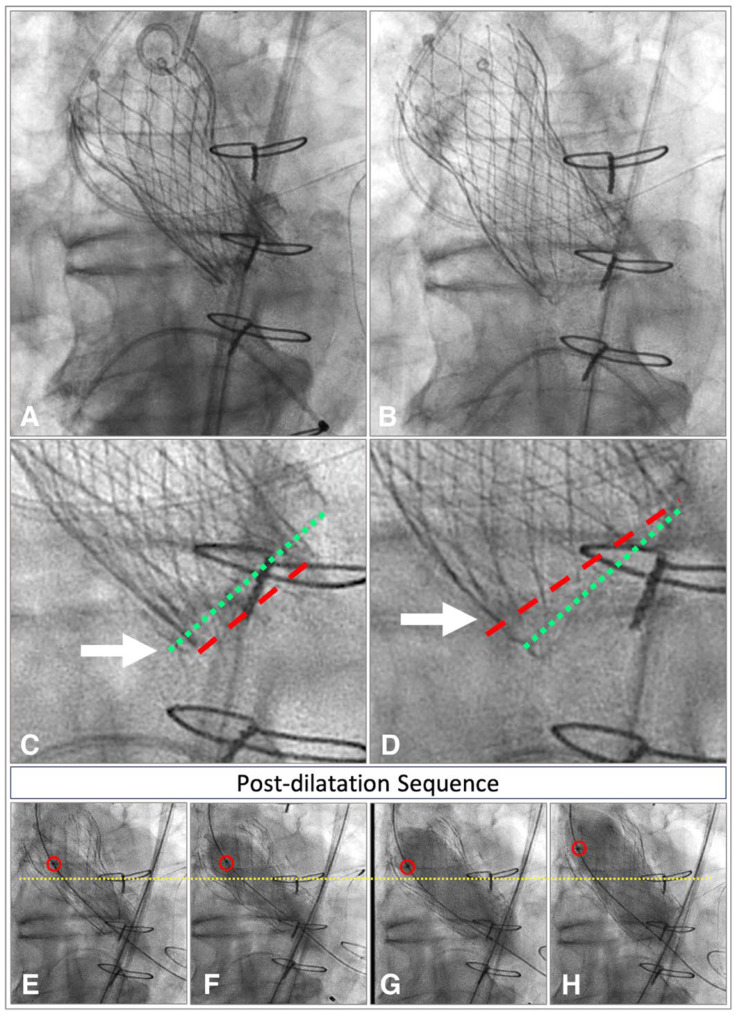
Angiographic view of a Corevalve Evolut R with “V” shape at deployment and its post-dilatation sequence during a valve-in-valve procedure. Corevalve Evolut R with a V shape at implantation (**A**) and after post-dilatation (**B**), before post-dilatation (**C**), the virtual basal ring (green dotted line) is located at a higher level compared to the inflow portion of the prosthesis (red dotted line); while after post-dilatation (**D**), upward movement of the prosthesis is observed, with an inversion of the relationship between the VBR (green dotted line) and the inflow portion of the prosthesis (red dotted line), which is displaced upwards; post-dilatation sequence (**E**–**H**) shows the upward displacement of the prosthesis, as evidenced by the movement of the proximal balloon marker (red circle) away from the reference point represented by the sternal tie (yellow dashed line).

**Figure 7:**
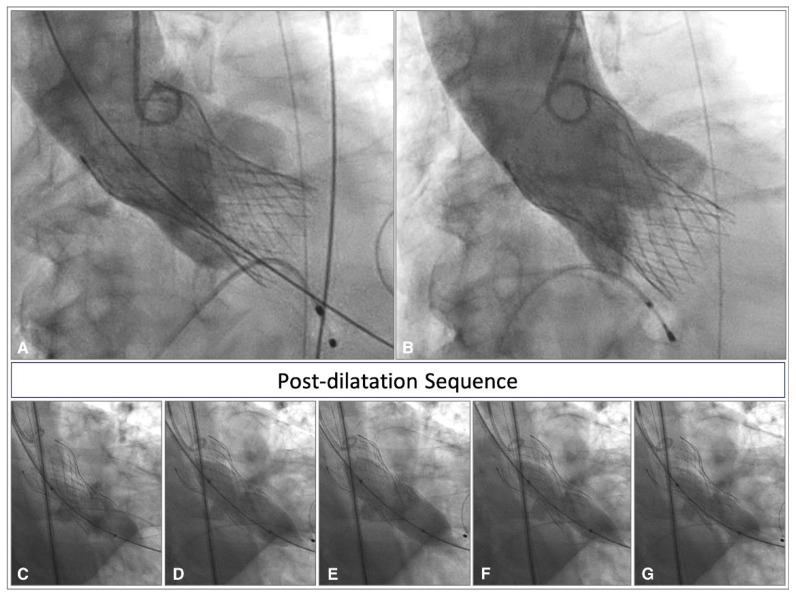
Angiographic view of a Corevalve Evolut R with “Bench” shape at deployment and its post-dilatation sequence in TAV anatomy. Corevalve Evolut R with a Bench shape (**A**) at deployment due to deep implantation; suboptimal post-dilatation result (**B**), retaining the same configuration; ineffective post-dilatation sequence (**C**–**G**) showing the recoil of the prosthesis and restoration of the Bench shape.

Indeed, both in BAV anatomy and in patients with TAV, we identified three different shapes of the prosthesis at deployment, each correlating with different risks of THV dislodgment during post-dilatation (Figure 4):

- Hourglass shape or “K shape” (in the case of only a one-sided neck): this shape is characterized by a constricted waist in the inflow portion of the THV, corresponding to the height of the raphe in BAV anatomies. This configuration benefits from two opposing forces that ensure the stability of the prosthesis, thereby minimizing the risk of embolization (Figure 5).

- Flared shape or “V shape”: This shape indicates a too high implantation depth and can strongly compromise the stability of the prosthesis, resulting in an elevated risk of embolization in case of post-dilatation because the forces acting on the prosthesis are directed upward (Figure 6).

- Cylindrical shape or “Bench shape”: This shape indicates a deeper implantation that increases the risk of residual aortic insufficiency, as it interferes with the proper sealing of the valve, allowing blood to flow directly from the prosthesis outflow into the LVOT during the diastolic phase. Moreover, in this configuration, the constricted waist corresponds to the narrowest point of the prosthesis as it was manufactured, thus will respond to post-dilatation with recoil and restoration of the Bench shape (Figure 7).

In the case of a “cylindrical” or “flared configuration,” a retrievable prosthesis that guarantees accurate repositioning by millimetric variation is desirable to guarantee an optimal implantation depth.

**Figure 8:**
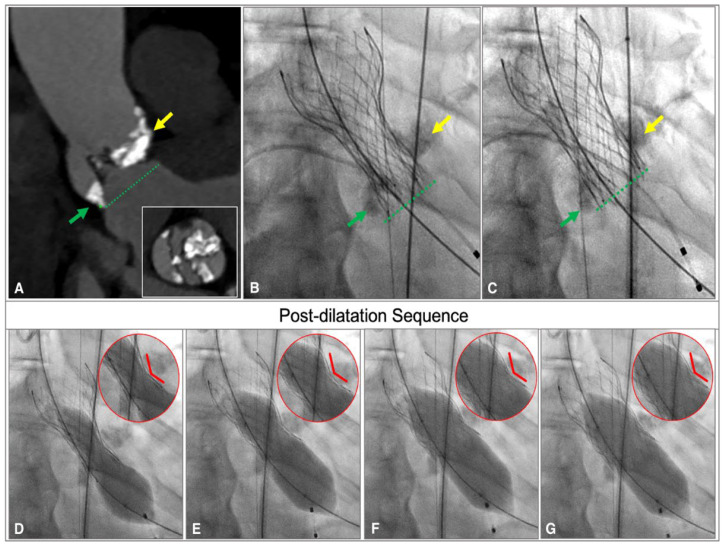
CT and Angiographic view of a Corevalve Evolut FX with a “K” shape at deployment and its post-dilatation sequence in BAV anatomy. Long-axis and short-axis CT scans show a raphe-type BAV where green arrow indicates VBR plane and yellow arrow indicates the LIRA plane (**A**); angiographic view of a Corevalve Evolut FX with two prevalent points of constraints corresponding to VBR plane (green arrow) and LIRA plane (yellow arrow) before pre-dilatation (**B**) and after post-dilatation (**C**); effective post-dilatation sequence (**D**–**G**) of a highly constrained prosthesis with persistence of the waist and the K shape (red circle).

This precision is even more crucial in patients with BAV, where anchoring is distributed across multiple anatomical planes (Figure 8).

Previous studies have shown that the new Evolut FX, due to the enhancements in the THV delivery system, has improved the coaxiality of the delivery catheter during deployment [13]. In agreement with these data, even in our series of patients with BAV, we observed that all THVs were implanted at the desired height and maintained a consistent depth and orientation throughout all the deployment phases (Figure 2). Indeed, the stability layer of the Evolut FX improves the prosthesis coaxially during deployment and leads to a more symmetrical and predictable implantation. This is particularly advantageous in patients with BAV, where valve anchoring is volumetric and depends on multiple anatomical structures. Additionally, the radiopaque markers in the Evolut FX frame are crucial in ensuring accurate implantation depth and facilitating commissural alignment.

Importantly, the delivery system has increased flexibility and trackability, enhanced by a smooth tip compared to previous generations that improves navigation within the vascular system. Indeed, even in our cohort of patients we did not observe any vascular complications.

## 5. Limitations

Our study has certain limitations inherent to its small sample size and the absence of a control group for outcome comparison.

Implantation depth and the decision to perform post-dilatation were left to the operators’ discretion.

Moreover, implantation depths were determined by aortography and subject to inherent methodological limitations.

## 6. Conclusions

In our study, the combination of the LIRA sizing method with the use of the Evolut FX in raphe-type patients with BAV undergoing TAVR yielded promising results. The Evolut FX’s enhancements allowed an accurate and stable THV deployment, with technical and device success achieved in all patients. Further multi-center studies with larger cohorts are necessary to validate these initial findings. Moreover, long-term evaluations will be decisive in assessing the durability of the Evolut FX prosthesis in the context of BAV.

## Figures and Tables

**Table 1:** Key technical features of the different evolutions of Medtronic Corevalve THV.

	Corevalve	Evolut R	Evolut Pro	Evolut Pro+	Evolut FX
**Size**	23-26-29-31	23-26-29-34	23-26-29	23-26-29-34	23-26-29-34
**Minimal Vessel Diameter**	≥6.0 mm	≥5.0 mm	≥5.5 mm	≥5.0 mm	≥5.0
**Technological improvements**	Not recapturable	-Recapturable -Sealing skirt	External pericardial wrap	PTFE inner capsule layer	-Gold markers -Stability layer

**Table 2:** Main studies underlying the differences between the Evolut Fx and the Evolut Pro+.

Study	Findings on Evolut FX	Comparison to Evolut PRO+
First-in-Human Multicenter Experience of the Newest Generation Supra-Annular Self-Expanding Evolut FX TAVR System. *JACC Cardiovasc Interv*. **2023**, *16*, 1626–1635. Zaid et al. [13]	96.5% commissural aligment. Symmetrical implant depth with LCC depth at 4.5 ± 2.1 mm. Low rate of PVL: 14.3% mild PVL 0.9% moderate PVL 0% severe PVL	Better commissural alignment - more symmetrical implant depth - fewer device recaptures. Improved PVL while maintaining similar hemodynamic performance.
Early experience with Evolut FX vs PRO+ for patients with aortic stenosis undergoing TAVR. *Cardiovasc Revasc Med*. **2023**, *56*, 1–6. Merdler et al. [14]	99 % VARC-3 technical success. 92% VARC-3 device success. 81.5% VARC-3 safety endpoint. 12% permanent pacemaker implantation.	No differences in clinical outcomes according to VARC-3 criteria.
Use and performance of the Evolut FX TAVR system. *Cardiovasc Revasc Med*. **2024**, *67*, 1–7. Bajwa et al. [15]	96.1 % commissural alignment. 80.6% valve coaxiality. 78.5% predictable deployment. 77.3% stable deployment. 35.9% valve recapture.	Better commissural alignment—valve coaxiality Superior deliverability More stable deployment

**Table 3:** Baseline clinical characteristics.

Clinical Characteristics	
Age (years)	(80.4 ± 4)
Male	5 (100%)
Arterial hypertension	5 (100%)
Diabetes mellitus	0 (0)
COPD	0 (0)
Peripheral artery disease	1 (20%)
Previous PCI or CABG	1 (20%)
Previous stroke	0 (0)
eGFR < 30 ml/min	2 (40%)
NYHA class ≥3	3 (60%)
STS risk score (%)	4 ± 2
1° grade AVB	1 (20%)
Right bundle branch block	2 (40%)
Left bundle branch block	1 (20%)

Values are expressed as mean ± standard deviation or n/N of patients (%). COPD = chronic obstructive pulmonary disease; PCI = percutaneous coronary intervention; CABG = coronary artery bypass graft surgery; eGFR = estimated glomerular filtration rate; NYHA = New York Heart Association; STS = Society of Thoracic Surgeons; AVB = atrioventricular block.

**Table 4:** Baseline echocardiographic findings.

Baseline Echocardiographic Findings	
Left ventricular ejection fraction (%)	52 ± 15
Aortic valve area (cm^2^)	0.7 ± 0.3
Mean aortic gradient (mmHg)	40.8 ± 9
Low flow- low gradient aortic stenosis	1 (20%)
Aortic regurgitation ≥moderate	2 (40%)

Values are expressed as mean ± standard deviation or n/N of patients (%).

**Table 5:** Baseline CT scan findings.

Baseline CT Scan Findings	
Type 1 BAV	5 (100)
Tapered configuration	5 (100%)
**Raphe location**	
Right/Left coronary	5 (100%)
Right/Non coronary	0 (0)
Left/Non coronary	0 (0)
**Raphe characteristics**	
Fibrotic	0 (0)
Calcific	5 (100)
**Annular sizing**	
Diameter min (mm)	26.5 ± 1.1
Diameter max (mm)	31.8 ± 1.9
Perimeter (mm)	93.7 ± 4.6
Ellipticity index	1.1 ± 0.1
**LIRA plane sizing**	
LIRA plane height from VBR (mm)	10.1 ± 3.3
Perimeter (mm), mean±SD	81 ± 4.9
Ascending aorta (mm)	39.3 ± 1.7
Horizontal aorta	3 (60)
Calcium score mm^3^	1912 ± 540.7
**Coronary height**	
Left main coronary artery (mm)	14 ± 1.7
Right coronary artery (mm)	18.8 ± 4.7

Values are expressed as mean ± standard deviation or n/N of patients (%). BAV = bicuspid aortic valve; VBR = virtual basal ring; SD = standard deviation.

**Table 6:** Procedural characteristics.

Procedural Characteristics	
Femoral access	5 (100%)
Predilatation	5 (100%)
Predilatation balloon size, median (IQR)	23 (20–24)
THV size	
Corevalve Evolut FX 23–26 mm	0 (0)
Corevalve Evolut FX 29 mm	2 (40%)
Corevalve Evolut FX 34 mm	3 (60%)
Post-dilatation	3 (60%)
Valve repositioning	0 (0)
Valve migration or second valve implantation	0 (0)

Values are expressed as median (inter-quartile range) or n/N of patients (%).

**Table 7:** 30-day outcomes.

30-Days Outcomes	
All cause death	0 (0)
Stroke	0 (0)
Permanent pacemaker	1 (20%)
Mean gradient (mmHg)	5.8 ± 2.3
Paravalvular leak ≥moderate	0 (0)
VARC-3 device success	5 (100%)
VARC-3 safety	4 (80%)

Values are expressed as mean ± standard deviation or n/N of patients (%). VARC = Valve Academic Research Consortium.

## Data Availability

Data are contained within the article.

## Abbreviations

(AVB)	Atrioventricular block
(BAV)	Bicuspid aortic valve
(BEV)	Balloon-expandable Valve
(CT)	Computer tomography
(EKG)	Electrocardiogram
(ECG)	Electrocardiography
(IQR)	Inter-quartile range
(LAO)	Left Anterior Oblique
(LCC)	Left coronary cusp
(LVEF)	Left Ventricular Ejection Fraction
(LIRA)	Level of Implantation at the Raphe
(NCC)	Non coronary cusp
(NYHA)	New York Heart Association
(PM)	Pacemaker
(PPI)	Permanent Pacemaker Implantation
(PVL)	Paravalvular leak
(RBBB)	Right bundle branch block
(SEV)	Self-expanding valve
(TAV)	Tricuspid aortic valve
(TAVI)	Transcatheter aortic valve implantation
(TAVR)	Transcatheter aortic valve replacement
(THV)	Transcatheter heart valve
(TTE)	Transthoracic echocardiography
(VARC3)	Valve Academic Research Consortium 3
(VBR)	Virtual basal ring
